# Antileukemic Activity of *Sechium* Hybrid H387 Extract Associated with SIRT1 Downregulation and p53 Acetylation in Human Chronic Myeloid Leukemia Cells

**DOI:** 10.3390/molecules31172998

**Published:** 2026-08-27

**Authors:** Itzen Aguiñiga-Sánchez, Daniel Romero-Trejo, Karen Miranda-Duarte, Ernesto Romero-López, Jorge Cadena-Iñiguez, Lorena Shira, Juana Rosado-Pérez, Víctor Manuel Mendoza-Núñez, Víctor Manuel Macías Zaragoza, Benny Weiss-Steider, Edelmiro Santiago-Osorio

**Affiliations:** 1 Hematopoiesis and Leukemia Laboratory, Research Unit on Cell Differentiation and Cancer, Faculty of High Studies Zaragoza, National Autonomous University of Mexico, Mexico City 09230, Mexico; liberitzen@comunidad.unam.mx (I.A.-S.); danielromero@unam.edu (D.R.-T.);; 2 Department of Biomedical Sciences, School of Medicine, Faculty of High Studies Zaragoza, National Autonomous University of Mexico, Mexico City 56410, Mexico; 3 Research Department, Bioplenum Biotechnology Institute, Puerto Vallarta 48313, Mexico; 4 Postgraduate College, Campus San Luis Potosí, Salinas de Hidalgo 78622, Mexico; 5 Research Unit on Gerontology, FES Zaragoza, National Autonomous University of Mexico, Mexico City 09230, Mexico

**Keywords:** chronic myeloid leukemia, imatinib, *Sechium* hybrid H387 extract, SIRT1, p53 acetylation, apoptosis

## Abstract

Chronic myeloid leukemia (CML) is driven by constitutive BCR–ABL1 activity and remains clinically challenging due to disease persistence and resistance to tyrosine kinase inhibitors (TKIs). SIRT1, a NAD^+^-dependent deacetylase, has been implicated in leukemic cell survival and chemoresistance through negative regulation of p53. In this study, we evaluated the antileukemic effects of the *Sechium* hybrid H387 extract and its impact on the SIRT1–p53 signaling axis in K562 CML cells. Cell proliferation was assessed using a crystal violet assay, while gene and protein expression were analyzed by RT-qPCR, immunofluorescence, and flow cytometry. Apoptosis was determined by Annexin V/7-AAD staining. The extract significantly inhibited K562 cell proliferation and downregulated SIRT1 expression, reducing its nuclear localization. These effects were associated with increased p53 acetylation at lysine 382 (Ac-K382-p53) and enhanced apoptosis. Notably, a higher percentage of apoptotic cells was observed following extract treatment than with imatinib under the experimental conditions used, although the two agents have distinct mechanisms of action. These findings suggest that the antileukemic activity of *Sechium* hybrid H387 extract is associated with modulation of the SIRT1–p53 axis and induction of apoptosis in K562 cells.

## 1. Introduction

Chronic myeloid leukemia (CML) is a clonal myeloproliferative disorder driven by the constitutive activity of the BCR-ABL1 tyrosine kinase [1]. CML typically presents in a chronic phase (CP), progresses to an accelerated phase (AP), and eventually culminates in blast crisis (BC), an aggressive stage associated with treatment resistance, chemoresistance, and disease recurrence [1,2]. Tyrosine kinase inhibitors (TKIs), such as imatinib, dasatinib, nilotinib, bosutinib, and ponatinib, effectively induce remission and prolong survival in CP-CML patients, but their efficacy is limited in advanced disease stages [3,4,5].

SIRT1, a NAD^+^-dependent deacetylase, has been implicated in cancer progression and chemoresistance across multiple malignancies, including breast, bladder, colon cancers, and leukemias such as acute myeloid leukemia and chronic myeloid leukemia (AML and CML [6,7,8,9,10]). Mechanistically, SIRT1 promotes oncogenic signaling through activation of the Akt, ERK1/2, and BCL-2 pathways [11,12,13,14], thereby enhancing cell survival and resistance to apoptosis. In addition, SIRT1 directly deacetylates key transcriptional regulators, including p53 [15], PTEN [16], Ku70 [17], and FoxO family members [18], leading to reduced tumor-suppressor activity, increased leukemic cell proliferation, and reduced sensitivity to chemotherapeutic agents. Overexpression of SIRT1 has been reported in CML blast crisis cell lines, highlighting it as a potential therapeutic target [19,20].

*Sechium edule* (Jacq.) Sw., commonly known as “chayote,” belongs to the Cucurbitaceae family and is widely consumed as part of the human diet. Traditional uses of *Sechium edule* include hypotensive, anti-inflammatory, and antioxidant applications [21,22], as well as benefits in kidney stone elimination, ulcer healing, and relief of intestinal disorders [21]. Importantly, previous studies have demonstrated that *Sechium edule* extracts exert selective antitumor activity in cervical cancer and acute myeloid leukemia (AML) cells without affecting healthy cells [23,24]. Among these bioactive preparations, a hybrid *Sechium* extract designated H-387-07-GISeM^®^, derived from the *Sechium edule* varietal group Wild Type II (chayote), has shown promising antiproliferative properties [25]. Previous phytochemical investigations of the *Sechium edule* H387 hybrid extract have provided a defined chemical profile that includes phenolic acids, flavonoids, and cucurbitacins [26,27]. HPLC analysis using authentic external standards identified and quantified eight flavonoids and eight phenolic acids [26], whereas subsequent HPLC and HPTLC analyses expanded the characterization to cucurbitacins, including cucurbitacin B as the predominant identified cucurbitacin [27]. Among the quantified constituents, galangin, phloretin, naringenin, and chlorogenic acid were prominent polyphenolic components [26]. Because the extract is a multicomponent botanical preparation, these previously established quantitative and chromatographic data provide an important chemical context for interpreting the biological activity observed in the present study and for future extract standardization [26,27]. However, the antileukemic potential and underlying molecular mechanisms of the *Sechium* hybrid H387 extract in human chronic myeloid leukemia (CML) cells remain largely unexplored. K562 cells are characterized by impaired p53 expression and function, and previous studies have reported either absent or undetectable p53 protein in this cell line [27,28]. Therefore, assessment of p53-related responses in K562 cells requires careful interpretation, particularly when evaluating post-translational modifications such as p53 acetylation. In this work, we investigated whether the antileukemic activity of *Sechium* hybrid H387 extract is associated with SIRT1 modulation, p53 acetylation, inhibition of proliferation, and induction of apoptosis in K562 CML cells.

## 2. Results

### 2.1. Basal Characterization of K562 Cell Line

**Figure 1:**
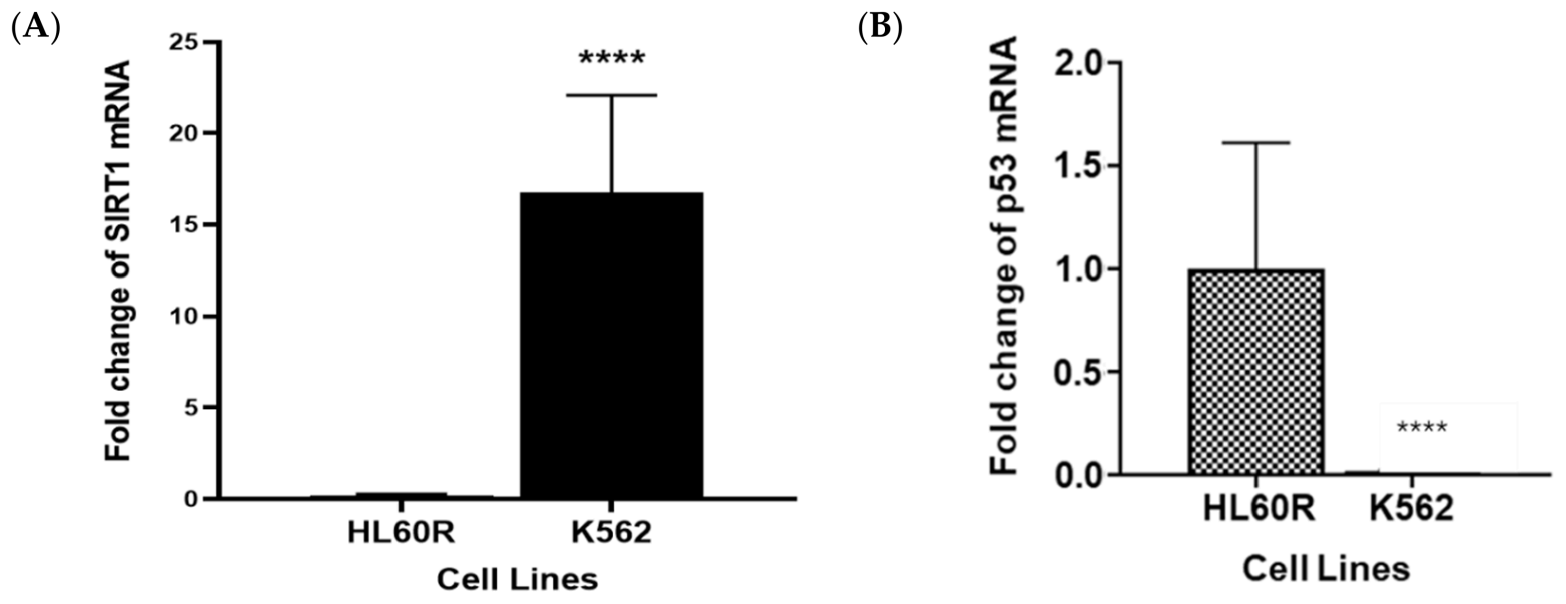
Molecular characterization of the human K562 chronic myeloid leukemia cell line. (**A**,**B**) Quantitative real-time RT-qPCR analysis of SIRT1 and p53 mRNA expression levels normalized to β-actin. Cytarabine-resistant HL-60 cells were used as a reference control. Data are presented as the mean ± SD from three independent experiments. Statistical significance was assessed using Student’s *t*-test (**** *p* < 0.0001).

To investigate the effects of the *Sechium* hybrid H387 extract on human K562 CML cells, we first evaluated the basal expression of SIRT1, a molecule implicated in cell proliferation and chemoresistance. In parallel, the expression of p53, a key tumor-suppressor gene, was analyzed in this cell line. Using real-time reverse transcription–polymerase chain reaction (RT-qPCR), we observed that K562 cells expressed SIRT1, whereas p53 expression was undetectable under basal culture conditions (Figure 1A,B), in contrast to the human acute myeloid leukemia cell line HL-60 used as a control. These findings indicate that the presence of SIRT1 and the lack of detectable basal p53 transcripts render K562 cells an appropriate experimental model for evaluating the effects of *Sechium* hybrid H387 extract. Consistently, previous reports have validated K562 cells as a representative CML model and have demonstrated p53 dysregulation in this cell line [28].

### 2.2. Antileukemic Effect of Sechium *Hybrid* H387 Extract on K562 Cell Proliferation

**Figure 2:**
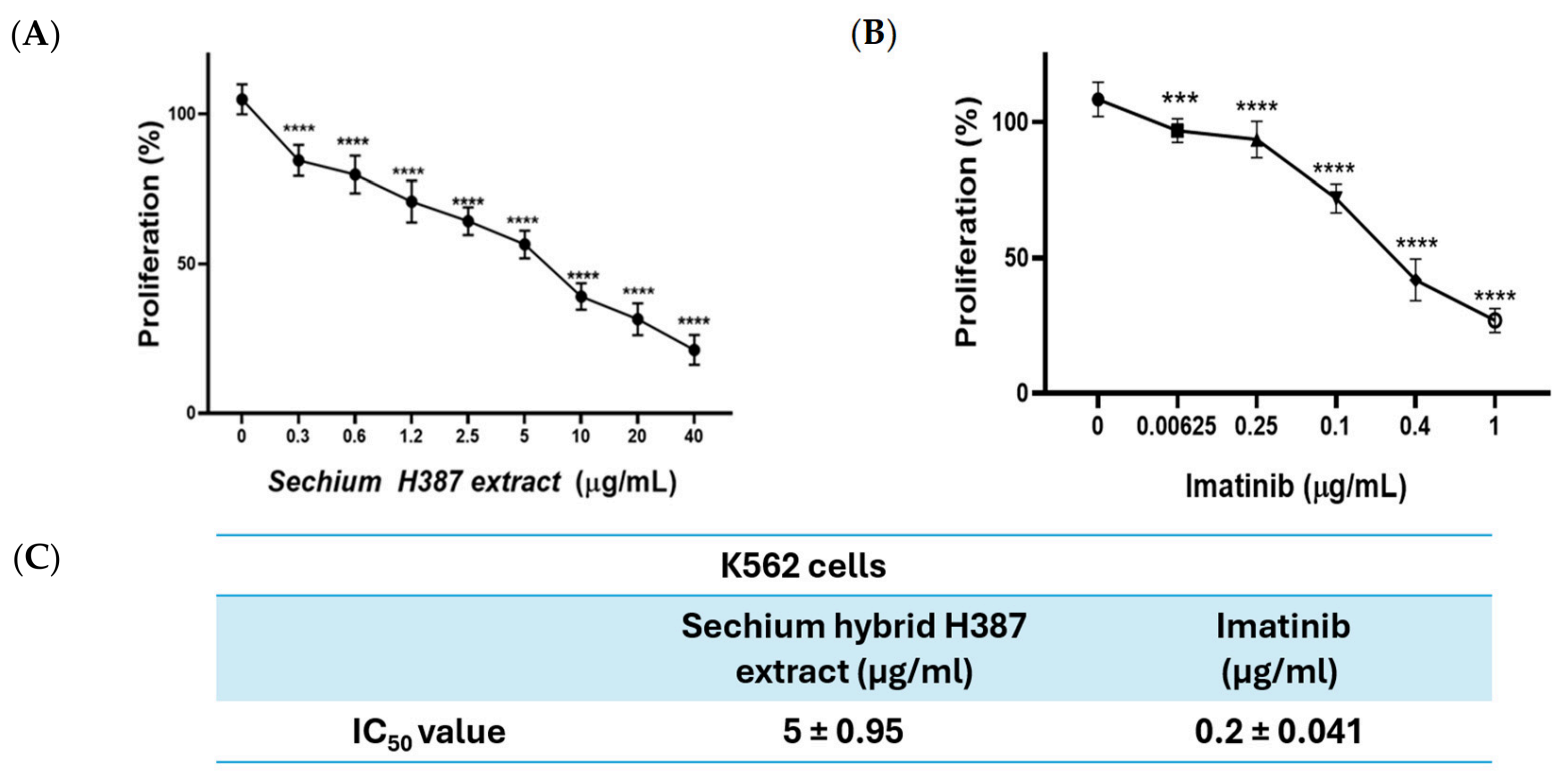
Effect of *Sechium* hybrid H387 extract and imatinib on K562 cell proliferation. (**A**,**B**) K562 cells were exposed to increasing concentrations of *Sechium* hybrid H387 extract or imatinib for 72 h, and cell proliferation was evaluated using the crystal violet assay. Data are expressed as the mean ± SD of triplicate determinations from three independent experiments. Statistical significance was assessed by one-way ANOVA followed by Dunnett’s post hoc test (*** *p* < 0.001; **** *p* < 0.0001). (**C**) IC_50_ values of *Sechium* hybrid H387 extract and imatinib in K562 cells.

To assess the antiproliferative effect of *Sechium* hybrid H387 extract, K562 cells were exposed to increasing concentrations of the extract, and cell proliferation was evaluated. Treatment with *Sechium* hybrid H387 extract resulted in a concentration-dependent inhibition of cell growth compared with PBS-treated controls (Figure 2A). In addition, a proliferation curve was generated using imatinib as a reference compound for subsequent experiments (Figure 2B). As shown in Figure 2C, IC_50_ values calculated using GraphPad Prism 8 were 5.0 ± 0.95 μg/mL for *Sechium* hybrid H387 extract and 0.20 ± 0.041 μg/mL for imatinib. The respective IC_50_ concentrations were used in subsequent experiments.

### 2.3. Phytochemical Context of the Tested Sechium *Hybrid* H387 Extract

The biological assays in the present study were performed using a previously characterized methanolic *Sechium* hybrid H387 07 extract. The phytochemical composition of this hybrid has been characterized in previous studies, including the identification and quantification of eight flavonoids and eight phenolic acids by HPLC [26], followed by subsequent characterization of cucurbitacins using HPLC and HPTLC [27]. The major quantified constituents relevant to the present study are summarized in Table 1.

### 2.4. Effect of Sechium *Hybrid* H387 Extract on Apoptosis Induction and p53 Acetylation in K562 Cells

**Figure 3:**
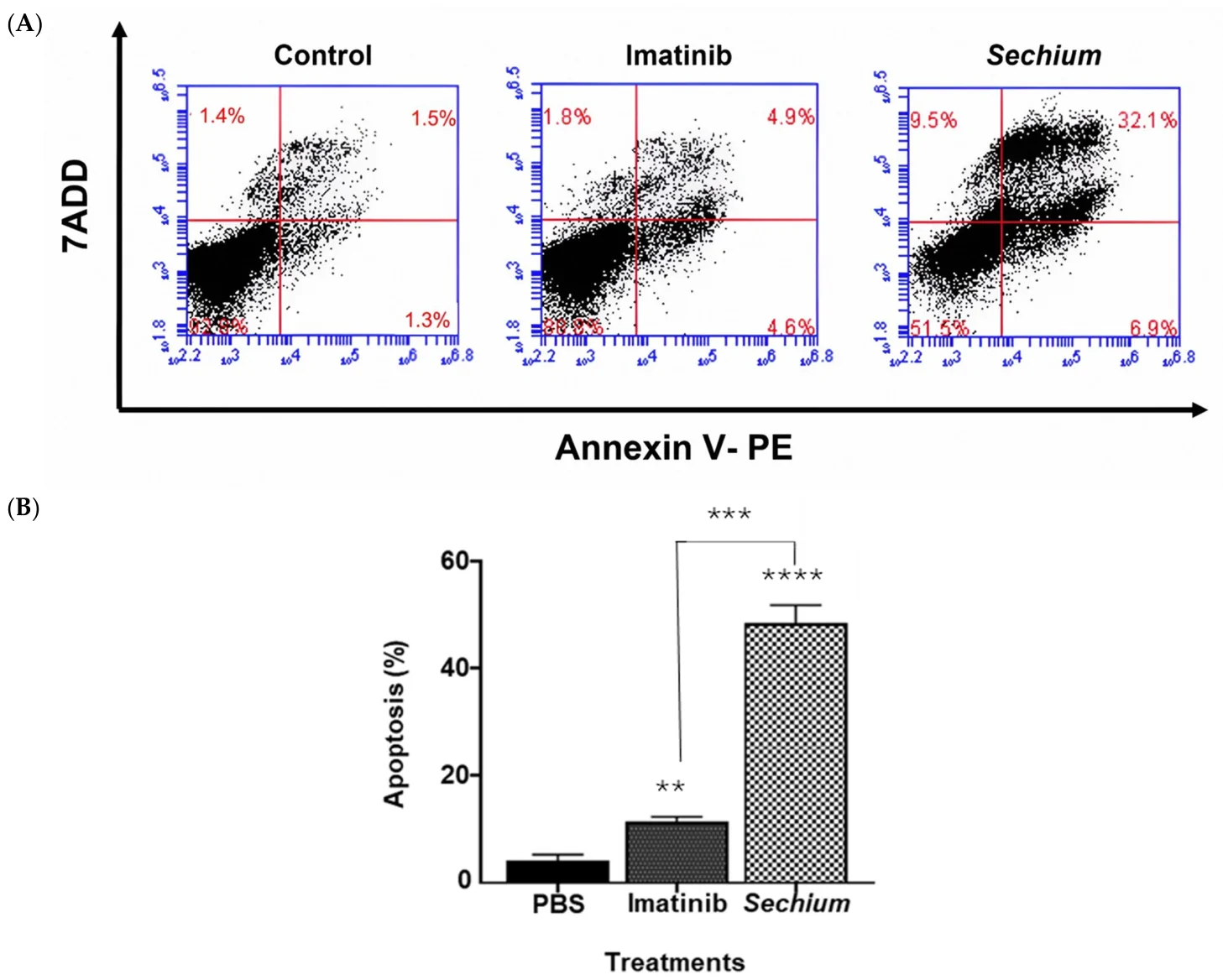
*Sechium* hybrid H387 extract induces apoptosis in K562 cells. (**A**) K562 cells were treated with the IC_50_ concentrations of *Sechium* hybrid H387 extract or imatinib for 48 h, and apoptosis was assessed by flow cytometry. Representative dot plots show Annexin V versus 7-AAD staining. (**B**) Percentage of apoptotic cells following the indicated treatments. Data represent the mean ± SD of three independent experiments. Statistical significance was analyzed using one-way ANOVA followed by Tukey’s post hoc test (** *p* < 0.01; *** *p* < 0.001; **** *p* < 0.0001).

To evaluate whether *Sechium* hybrid H387 extract was associated with apoptosis induction, Annexin V-based flow cytometry analysis was performed. After 48 h of treatment, *Sechium* hybrid H387 extract induced apoptosis in approximately 45% of K562 cells, whereas imatinib induced apoptosis in approximately 12% of cells at their respective IC_50_ concentrations (Figure 3A,B). The apoptotic response induced by *Sechium* hybrid H387 extract was approximately 3.2-fold higher than that observed with imatinib (Figure 3B). These findings indicate that *Sechium* hybrid H387 extract was associated with a marked increase in apoptosis under the experimental conditions used.

**Figure 4:**
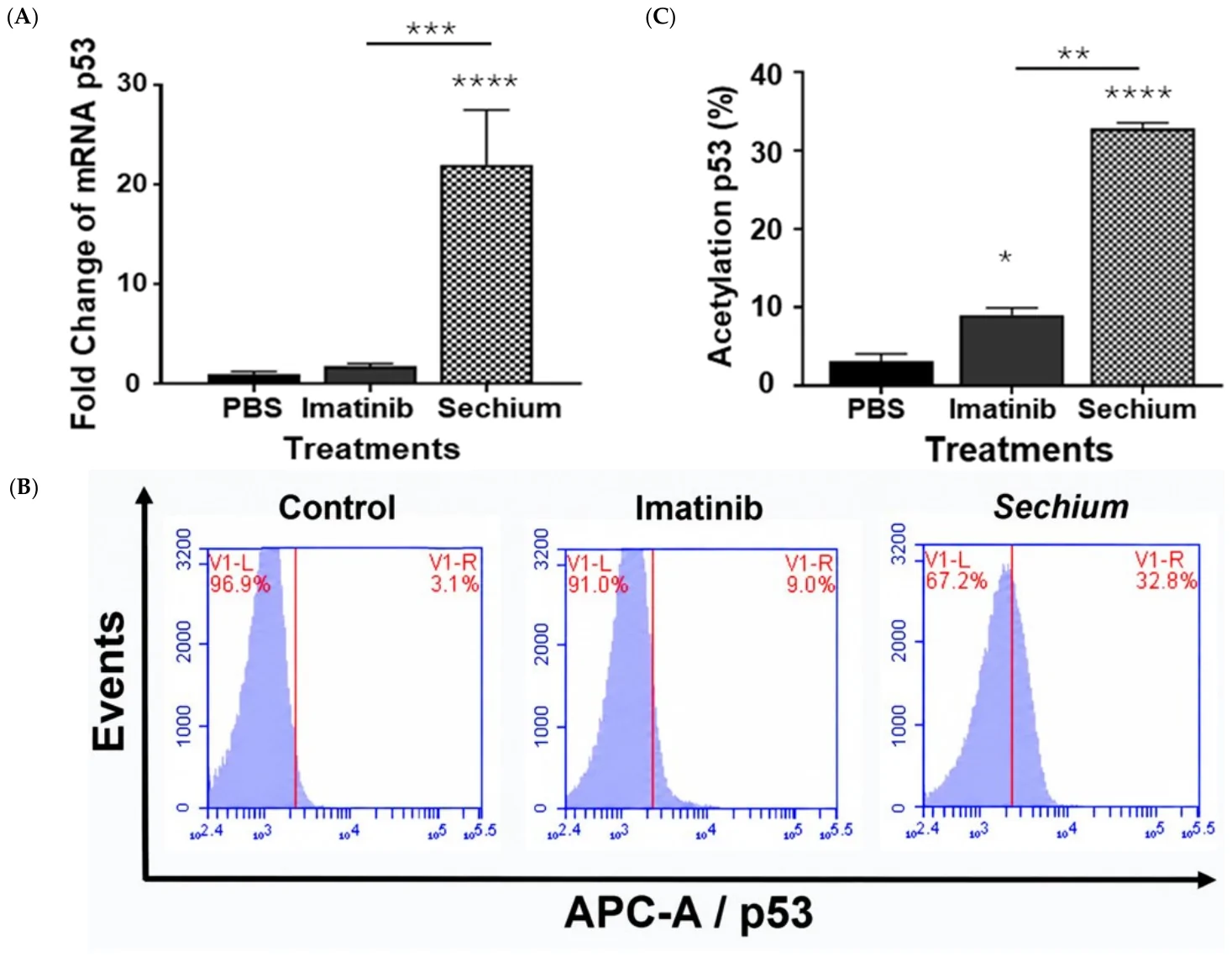
*Sechium* hybrid H387 extract increases p53 mRNA expression and p53 acetylation at lysine 382 in K562 cells. (**A**) Quantitative real-time RT-qPCR analysis of p53 mRNA expression after 48 h of treatment with IC_50_ concentrations of *Sechium* hybrid H387 extract or imatinib. Data are expressed as fold change relative to untreated control and presented as the mean ± SD of three independent experiments. β-actin was used as the housekeeping gene, and relative expression was calculated using the 2^−ΔΔCT^ method. (**B**) Flow cytometric analysis of p53 acetylation at lysine 382 (AcK382) after the indicated treatments. Representative histograms show the distribution of events according to APC-A fluorescence corresponding to p53 (AcK382). (**C**) Percentage of p53 acetylation at K382 following the indicated treatments. Data represent the mean ± SD of three independent experiments. Statistical significance was determined using one-way ANOVA followed by Tukey’s post hoc test (* *p* < 0.05; ** *p* < 0.01; *** *p* < 0.001; **** *p* < 0.0001).

SIRT1-mediated deacetylation of p53 has been associated with impaired apoptotic responses in leukemia cells, suggesting that reduced p53 acetylation contributes to resistance to programmed cell death [28]. To assess whether treatment with *Sechium* hybrid H387 extract was associated with changes in p53 transcript levels and acetylation, K562 cells were analyzed after 48 h by RT-qPCR and flow cytometry. Treatment with *Sechium* hybrid H387 extract significantly increased p53 mRNA levels from the undetectable basal levels observed in untreated K562 cells (Figure 4A) and increased p53 acetylation at lysine 382 (Ac-K382-p53) (Figure 4B,C), resulting in an approximately 2.6-fold increase in Ac-K382-p53 signal compared with imatinib-treated cells.

### 2.5. Effect of Sechium *Hybrid* H387 Extract on SIRT1 Downregulation

**Figure 5:**
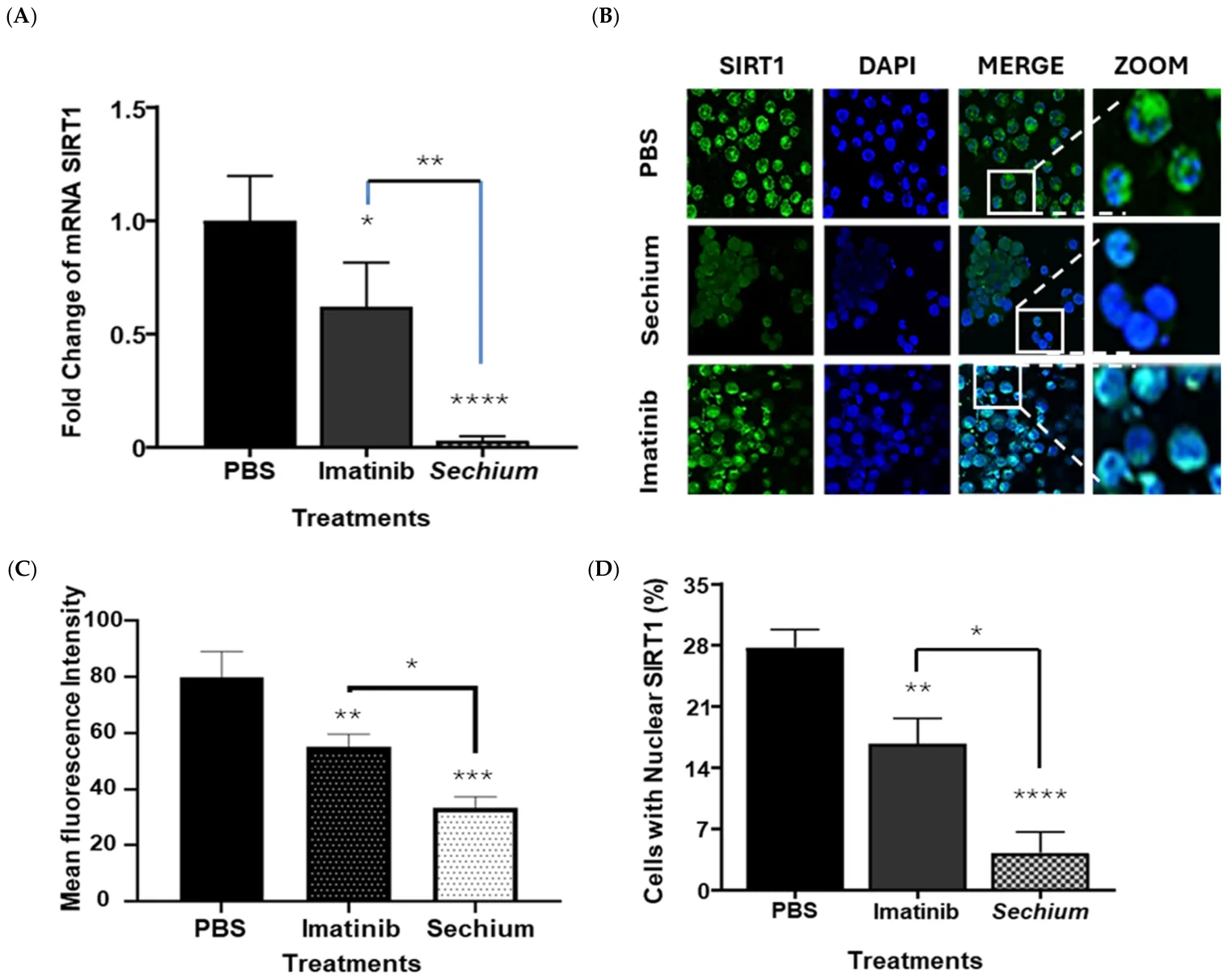
*Sechium* hybrid H387 extract downregulates the expression and nuclear localization of SIRT1 in K562 cells. (**A**) Quantitative real-time RT-qPCR analysis of SIRT1 mRNA expression in K562 cells treated with IC_50_ concentrations of *Sechium* hybrid H387 extract or imatinib for 48 h. Data are expressed as fold change relative to untreated control and presented as the mean ± SD of three independent experiments. β-actin was used as the housekeeping gene, and relative expression was calculated using the 2^−ΔΔCT^ method. (**B**) Confocal images showing SIRT1 staining (left), nuclei stained with DAPI (middle), and merged images (right). Scale bar: 50 µm. (**C**) Quantification of SIRT1 protein levels based on mean fluorescence intensity measured across three fields per experimental condition. (**D**) Quantification of SIRT1 nuclear localization based on mean values from three fields per experimental condition. Data represent three independent experiments and are expressed as mean ± SD. Statistical significance was determined using one-way ANOVA followed by Tukey’s post hoc test (* *p* < 0.05; ** *p* < 0.01; *** *p* < 0.001; **** *p* < 0.0001).

Overexpression of the deacetylase SIRT1 has been associated with enhanced proliferation, chemoresistance, and reduced apoptosis in CML cells [29]. To determine whether *Sechium* hybrid H387 extract modulates SIRT1 expression, K562 cells were treated for 48 h with *Sechium* hybrid H387 extract or imatinib, and SIRT1 levels were assessed by RT-qPCR and immunofluorescence. Both treatments significantly reduced SIRT1 expression compared with control cells; however, *Sechium* hybrid H387 extract produced a more pronounced reduction relative to imatinib-treated cells (Figure 5A,D). Immunofluorescence analysis further revealed that *Sechium* hybrid H387 extract markedly reduced SIRT1 expression and nuclear localization, decreasing the proportion of SIRT1-positive nuclei by more than 80% compared with all other experimental groups (Figure 5B,D). These findings indicate that *Sechium* hybrid H387 extract markedly reduced SIRT1 expression and nuclear localization under the experimental conditions used.

**Figure 6:**
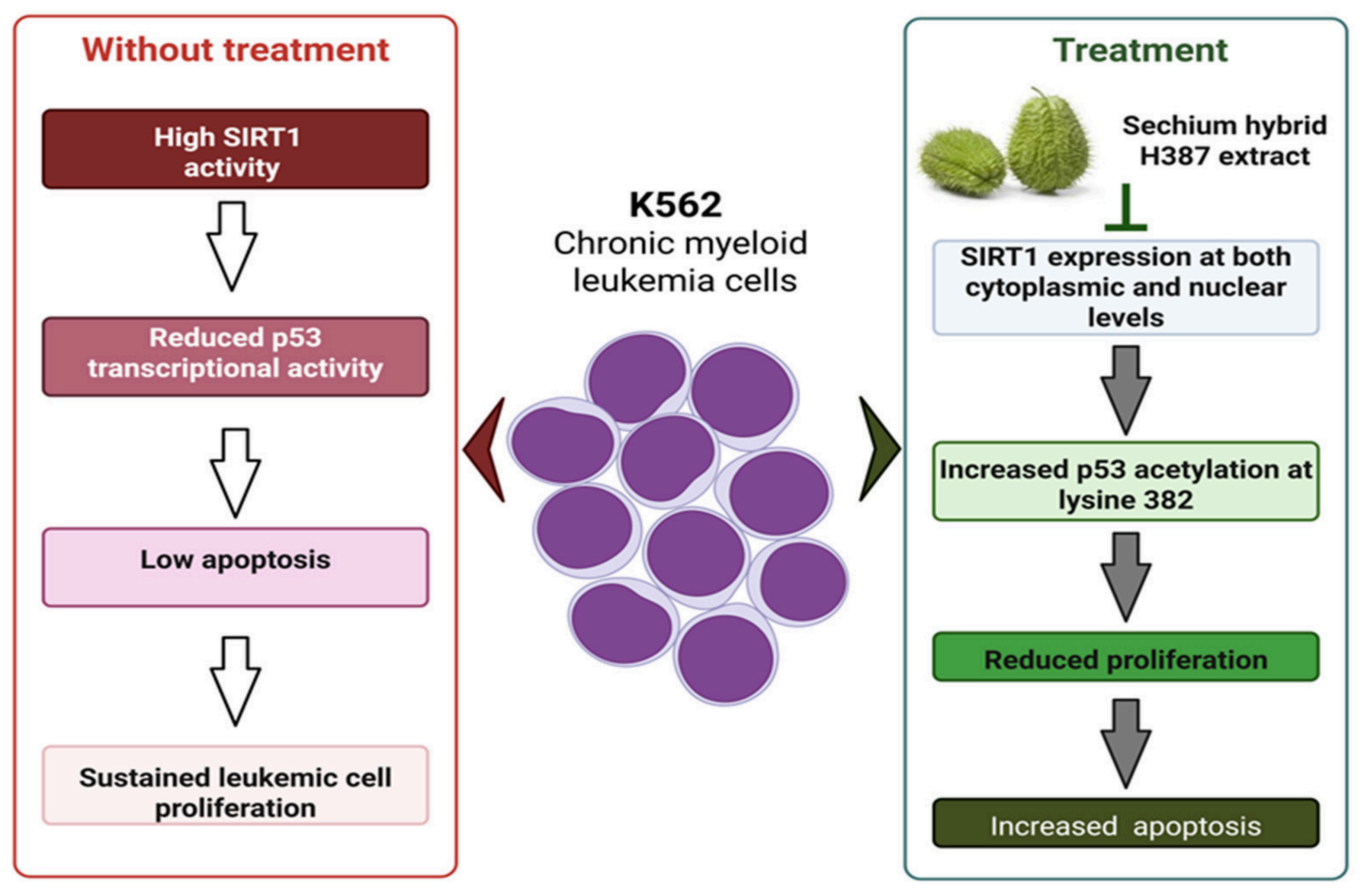
Proposed model illustrating the potential mechanism of action of *Sechium* hybrid H387 extract in chronic myeloid leukemia K562 cells. Treatment with *Sechium* hybrid H387 extract is associated with reduced K562 cell proliferation, decreased SIRT1 expression, increased p53 acetylation, and induction of apoptosis. These findings support a potential association between SIRT1 downregulation, increased p53 acetylation, and apoptotic cell death, which may contribute to the attenuation of leukemic cell growth.

In summary, treatment with *Sechium* hybrid H387 extract inhibited proliferation and induced apoptotic cell death in human CML cells and was associated with reduced SIRT1 expression, increased p53 mRNA levels, and increased p53 acetylation at lysine 382 (Figure 6).

## 3. Discussion

Chronic myeloid leukemia represents a paradigm of successful targeted therapy; nevertheless, disease persistence and the emergence of resistance remain significant clinical challenges [1,2]. In this study, we demonstrate that *Sechium* hybrid H387 extract reduces proliferation and induces apoptosis in K562 CML cells, accompanied by decreased SIRT1 expression and nuclear localization and increased p53 mRNA expression and acetylation at lysine 382 (AcK382). These findings are consistent with the established role of SIRT1 as a NAD^+^-dependent deacetylase that negatively regulates p53 activity through deacetylation and with previous evidence linking SIRT1 inhibition to p53 acetylation and apoptosis in leukemia cells [29]. Similarly, our previous work showed that sodium caseinate increased p53 acetylation and promoted apoptosis in chemoresistant promyelocytic leukemia cells [9]. Our findings are consistent with these previous observations, as H387 treatment simultaneously reduced SIRT1 expression and increased p53 acetylation at K382, suggesting that reduced SIRT1 expression may contribute to the apoptotic response observed in K562 cells. Nevertheless, the present study demonstrates an association between these molecular changes rather than a direct causal relationship. Functional experiments using selective SIRT1 inhibition, SIRT1 rescue, or purified constituents of the extract will be required to determine whether SIRT1 is a direct molecular target of H387.

Accumulating evidence indicates that plant-derived extracts and bioactive natural compounds can modulate sirtuin activity and restore p53-dependent apoptosis in cancer cells, including CML and AML [9,30]. Nutritional and bioactive compounds, particularly polyphenols and flavonoids, have been shown to regulate SIRT1-dependent pathways associated with apoptosis and cancer cell survival [24,31]. Furthermore, several extracts and phytochemicals evaluated in cancer models have demonstrated the capacity to downregulate SIRT1 and promote p53-mediated apoptosis, supporting the biological plausibility of our findings [23]. In this context, the reduction in SIRT1 observed after H387 treatment is biologically relevant because increased SIRT1 expression has been associated with leukemic cell survival, chemoresistance, and maintenance of CML stem-cell properties [28,29,30,31,32,33]. Thus, the concomitant decrease in SIRT1 and increase in p53 acetylation observed in the present study are consistent with a cellular response in which reduced deacetylase activity could improve p53 activation and apoptosis.

From a phytochemical perspective, H387 is a chemically complex extract rather than a single active substance. Previous HPLC characterization using authentic external standards identified eight flavonoids and eight phenolic acids, including rutin, phlorizin, myricetin, quercetin, naringenin, phloretin, galangin, apigenin, and several phenolic acids [26]. Among the quantified compounds, galangin was the most abundant identified flavonoid (21.940 mg/g extract), followed by phloretin (4.616 mg/g) and naringenin (3.304 mg/g), whereas chlorogenic acid was the predominant identified phenolic acid (4.224 mg/g extract) [26]. Additional characterization of H387 07 identified cucurbitacins, with cucurbitacin B being the predominant compound (4.595 mg/g extract), followed by cucurbitacin I (3.061 mg/g) and cucurbitacin E (0.594 mg/g), for a total quantified cucurbitacin content of 8.251 mg/g extract [27]. This quantitative profile provides a chemical context for the biological activity observed in K562 cells. Both polyphenols and cucurbitacins may contribute to the activity of the extract; however, their individual contributions cannot be distinguished with the present experimental design. In particular, cucurbitacin B has been reported to inhibit proliferation and induce apoptosis in K562 cells and to modulate STAT3 and Raf/MEK/ERK signaling [34]. Nevertheless, these findings do not establish that cucurbitacin B, galangin, or any other individual constituent mediates the SIRT1–p53 response induced by H387. Because the extract also contains additional unidentified chromatographic components [26], the observed activity may reflect the combined effects of multiple constituents, including possible additive or synergistic interactions.

At the IC_50_ concentration used in the biological experiments (5.0 ± 0.95 μg/mL extract), the reported phytochemical composition corresponds to estimated nominal concentrations of approximately 0.110 μg/mL galangin, 0.023 μg/mL cucurbitacin B, 0.015 μg/mL cucurbitacin I, 0.003 μg/mL cucurbitacin E, 0.023 μg/mL phloretin, 0.021 μg/mL chlorogenic acid, and 0.017 μg/mL naringenin. These values are theoretical concentrations calculated from the reported amounts per gram of extract and should not be interpreted as intracellular, free, or bioavailable concentrations. Therefore, they provide chemical context but cannot be used to assign the observed biological effects to individual compounds.

In addition to reducing overall SIRT1 expression, H387 treatment markedly decreased SIRT1 nuclear localization in K562 cells. This observation is relevant because nuclear SIRT1 participates in the regulation of several transcriptional and tumor-suppressor pathways, including p53, FoxO, and other factors involved in cellular survival and stress responses [15,18,32]. However, our immunofluorescence experiments demonstrate reduced nuclear localization of SIRT1 after treatment and do not directly demonstrate a change in the molecular process of SIRT1 nuclear translocation. Therefore, this finding should be interpreted as an alteration in SIRT1 subcellular distribution rather than as definitive evidence of inhibition of nuclear translocation. Further mechanistic studies will be required to determine whether changes in SIRT1 localization contribute independently to the apoptotic response induced by H387. Multiple studies have indicated that elevated SIRT1 expression is associated with poor molecular response, disease persistence, and resistance to tyrosine kinase inhibitors in CML [29,30,33,35]. These data support the role of SIRT1 as a survival factor in leukemic cells. In this context, our results provide experimental evidence that H387 treatment is associated with reduced SIRT1 expression and nuclear localization, together with increased p53 acetylation and apoptosis. These observations suggest that the SIRT1–p53 axis may contribute to the antileukemic response induced by the extract. Nevertheless, the term “targeting” should be interpreted cautiously because the present study did not determine whether SIRT1 is a direct molecular target of H387 or whether its downregulation is secondary to other cellular effects of the extract. Previous work from our group also demonstrated preferential activity of the H-387 extract toward leukemic cells, with strong inhibition of proliferation and induction of apoptosis in P388, J774, and WEHI-3 cells, whereas normal mouse bone marrow mononuclear cells were not affected under the experimental conditions used [23]. These findings provide supportive evidence for a differential response between leukemic and normal hematopoietic cells; however, this selectivity was not directly evaluated in the present K562 model. However, these previous observations should not be interpreted as evidence that the same individual metabolites are responsible for the effects observed in the present K562 model. The complex composition of botanical extracts makes it particularly important to distinguish extract-level activity from compound-level mechanisms. Strategies that induce apoptosis selectively in cancer cells without harming normal cells are essential for improving treatment outcomes in CML.

Another important consideration is extract standardization. In the present study, all biological experiments were performed using the same prepared batch of H387 extract, thereby reducing within-study variability. However, this does not constitute formal evidence of batch-to-batch reproducibility. Botanical extracts can vary according to plant genotype, developmental stage, environmental conditions, processing, extraction, storage, and analytical procedures. Current recommendations therefore emphasize detailed documentation of starting material and reproducible phytochemical characterization [36]. For H387, future batch-release specifications should combine extraction yield, chromatographic fingerprinting, and quantitative marker compounds such as galangin, chlorogenic acid, and cucurbitacin B. Stability studies should also determine whether extraction and storage conditions alter the concentration of these metabolites. A limitation of the present study is that a new HPLC/UHPLC fingerprint was not generated from the exact biological batch used in the experiments; instead, chemical characterization was based on our previous analyses of H387 material [26,27]. Although these studies provide quantitative information on several major constituents, they do not establish complete metabolomic coverage or formal inter-batch equivalence. Future studies should therefore include batch-specific chromatographic fingerprinting, preferably supported by HPLC–MS or UHPLC–MS/MS, together with quantitative marker analysis and predefined acceptance ranges to strengthen chemical traceability and reproducibility.

Overall, the present findings demonstrate that the *Sechium* hybrid H387 extract inhibits proliferation and promotes apoptosis in K562 CML cells, accompanied by reduced SIRT1 expression and nuclear localization and increased p53 mRNA expression and acetylation at lysine 382. The phytochemical profile of H387 provides a plausible chemical context for these effects, including the presence of abundant flavonoids and phenolic acids together with cucurbitacins such as cucurbitacin B. However, the current evidence supports an extract-level biological effect rather than attribution to a specific metabolite. The relationship between individual constituents and the SIRT1–p53 pathway remains to be established experimentally. Although our findings support an association between SIRT1 downregulation, increased p53 acetylation, and apoptosis, the present study does not establish a direct causal relationship among these events. Therefore, the SIRT1–p53 axis depicted in Figure 6 should be considered a proposed model rather than a validated signaling pathway. Future studies combining batch-specific chromatographic fingerprinting, activity-guided fractionation, purified-compound testing, and mechanistic rescue experiments will be necessary to identify the constituents responsible for SIRT1 modulation and to determine whether their activities are additive or synergistic.

## 4. Materials and Methods

### 4.1. Cell Culture

The human chronic myeloid leukemia K562 cell line (ATCC^®^ CCL-243™) was obtained from the American Type Culture Collection (Manassas, VA, USA). Cells were maintained in Iscove’s Modified Dulbecco’s Medium (IMDM; Gibco, Grand Island, NY, USA) supplemented with 10% fetal bovine serum (FBS; Gibco, Grand Island, NY, USA), 100 U/mL penicillin, and 100 μg/mL streptomycin (Gibco, Grand Island, NY, USA). Cultures were kept at a density of 1 × 10^5^ cells/mL in a humidified incubator at 37 °C with 95% air and 5% CO_2_ and were reseeded every 48 h.

### 4.2. Preparation and Characterization of Sechium *Hybrid* H-387 Extract

Fruits from the *Sechium* hybrid H387 07 were obtained from the National Germplasm Bank of Sechium in Mexico (19° 08′ 48″ N and 97° 57′ 00″ W), [35]. Fruits were harvested at horticultural maturity, 18 ± 2 days after anthesis [21]. The fruits were washed, dried, weighed, and cut into slices including the exocarp, mesocarp, spines, and seeds. The slices were dried in an oven at 40 °C with air circulation until completely dehydrated and were subsequently ground to a particle size of 2 mm. Then, 1.5 kg of powdered material was immersed in methanol for 48 h at room temperature (20 ± 2 °C). The solvent was recovered by filtration through Whatman No. 1 filter paper and evaporation under reduced pressure at 45 °C. This extraction cycle was repeated 25 times until the solvent showed no visible coloration [37,38]. The resulting methanolic extract was stored in amber vials until use and diluted in phosphate-buffered saline (PBS) immediately before the biological assays; hereafter, this material is referred to as the *Sechium* hybrid H387 extract.

The phytochemical characterization of the H387 07 extract has been previously reported [26,27]. Briefly, HPLC analysis using authentic external standards identified and quantified eight flavonoids (rutin, phlorizin, myricetin, quercetin, naringenin, phloretin, galangin, and apigenin) and eight phenolic acids (gallic, chlorogenic, syringic, vanillic, p-hydroxybenzoic, caffeic, ferulic, and p-coumaric acids). Quantification was based on calibration curves generated from the corresponding analytical standards. The principal quantified constituents included galangin (21.940 mg/g extract), phloretin (4.616 mg/g), chlorogenic acid (4.224 mg/g), naringenin (3.304 mg/g), rutin (1.273 mg/g), and myricetin (0.889 mg/g) [26]. A subsequent HPLC analysis of H387 material identified cucurbitacin B (4.595 mg/g extract), cucurbitacin I (3.061 mg/g), and cucurbitacin E (0.594 mg/g), corresponding to a total quantified cucurbitacin content of 8.251 mg/g extract. HPTLC provided independent chromatographic evidence for the presence of cucurbitacins, including cucurbitacin B [27]. Compound identification in the HPLC analysis was based on chromatographic comparison with authentic standards and quantitative calibration curves; therefore, these compounds are referred to as HPLC-identified constituents rather than compounds structurally confirmed by mass spectrometry. The previous analytical study also reported additional chromatographic peaks that could not be assigned because corresponding standards were unavailable. Accordingly, the compounds listed above represent characterized constituents and not the complete metabolome of the extract [26].

All biological experiments reported in the present study were performed using the same prepared batch of *Sechium* hybrid H387 extract. This approach minimized variability associated with extract preparation within the present study. However, because an independent inter-batch chromatographic validation study was not performed, batch-to-batch equivalence cannot be considered experimentally established. We therefore regard chromatographic fingerprinting together with quantitative marker compounds as an important component of future extract standardization. Candidate markers based on the present phytochemical evidence include galangin, chlorogenic acid, phloretin, and cucurbitacin B.

### 4.3. Proliferation Assay

K562 cells (5 × 10^5^ cells/mL) were treated in triplicate with increasing concentrations of *Sechium* hybrid H387 extract (0–40 μg/mL) or imatinib (0–500 ng/mL) for 72 h. PBS-treated cells served as control. Cell proliferation was measured using a crystal violet assay, and IC_50_ values were calculated. Absorbance was measured at 570 nm (Bio-Rad microplate reader, Hercules, CA, USA).

### 4.4. Real-Time RT-qPCR

Cells treated or untreated with IC_50_ concentrations of *Sechium* hybrid H387 extract or imatinib for 48 h were processed for RNA extraction (TRIzol, Invitrogen, Waltham, MA, USA). cDNA synthesis and RT-qPCR were performed using SYBR Green (Applied Biosystems, Thermo Fisher Scientific, Waltham, MA, USA) under 40 cycles (95 °C 10 s, 60 °C 30 s, 72 °C 15 s). Primers: SIRT1: forward, 5′-GCT GGA ACA GGT TGC GGG AA-3′; reverse, 5′-GGG CAC CTA GGA CAT CGA GGA-3′; p53: forward, 5′-GTT CCG AGA GCT GAA TGA GG-3′; reverse, 5′-TCT GAG TCA GGC CCT TCT GT-3′; β-actin: forward, 5′-CAC TGT CGA GTC GCG TCC-3′; reverse, 5′-CGC AGC GAT ATC GTC ATC CA-3′. Relative gene expression levels were calculated using the 2^−ΔΔCT^ method [38], with normalization to the β-actin housekeeping gene.

### 4.5. Immunofluorescence

K562 cells treated with IC_50_ concentrations of *Sechium* hybrid H387 extract or imatinib for 48 h were fixed with 4% paraformaldehyde, permeabilized (0.25% Triton X-100), and blocked with 1% BSA. Cells were incubated overnight at 4 °C with Alexa Fluor^®^ 488 mouse anti-SIRT1 antibody (1:200, Abcam, Cambridge, UK). The following day, the cells were rinsed three times with PBS and counterstained with 4′,6 diamidino-2-phenylindole (DAPI; Vector Laboratories, Newark, CA, USA) to visualize the nuclei. Images were captured using an inverted confocal microscope equipped with a 40× objective (TCS-SP2, Leica, Heidelberg, Germany). For each experimental condition, fluorescence intensity was quantified at the single-cell level within each field. A total of 80–100 cells per treatment group were analyzed from three randomly selected fields in three independent experiments.

### 4.6. Acetylated p53 Assay

K562 cells were seeded at a density of 3.2 × 10^4^ cells/mL in 60 mm culture dishes and treated with IC_50_ concentrations of *Sechium* hybrid H387 extract or imatinib, or left untreated for 48 h. Cells were harvested, washed twice with PBS, and 5 × 10^5^ cells were fixed and permeabilized using ice-cold BD Cytofix/Cytoperm™ solution (BD Biosciences, Piscataway, NJ, USA) for 20 min, followed by two washes with 1× BD Perm/Wash™ buffer. Cells were then incubated in the dark with an Alexa Fluor^®^ 647-conjugated mouse monoclonal anti-p53 antibody (BD Phosflow™, BD Biosciences, Piscataway, NJ, USA; Cat. No. 560231; clone L82-51), which specifically recognizes p53 acetylated at lysine 382 (Ac-K382). After 30 min, samples were washed and analyzed using a BD FACSAria™ II flow cytometer (BD Biosciences, Piscataway, NJ, USA). The fluorescence signal was used to assess the relative levels of p53 acetylated at lysine 382 (Ac-K382-p53).

### 4.7. Apoptosis Assay

K562 cells were seeded in 60 mm culture dishes at a density of 3.2 × 10^4^ cells/mL and treated with IC_50_ concentrations of *Sechium* hybrid H387 extract or imatinib, or left untreated. After 48 h, cells were collected, washed twice with PBS, and resuspended in 1× binding buffer at 1 × 10^5^ cells/mL. Cells were stained with Annexin V–PE/7-AAD (Becton Dickinson, Franklin Lakes, NJ, USA) for 15 min at room temperature in the dark. Subsequently, 1× Annexin V binding buffer was added, and samples were analyzed using a BD FACSAria™ II flow cytometer (BD Biosciences, San Jose, CA, USA) at 528 nm and 650 nm for PE and 7-AAD detection, respectively.

### 4.8. Statistical Analysis

Data are expressed as the mean ± standard deviation (SD) from three independent experiments. Statistical analyses were performed using one-way analysis of variance (ANOVA) followed by Tukey’s multiple comparison test or Student’s *t*-test, as appropriate. Statistical significance was defined as follows: * *p* < 0.05, ** *p* < 0.01, *** *p* < 0.001, and **** *p* < 0.0001 compared with individual treatments or between groups. All analyses were conducted using GraphPad Prism version 8.0 (GraphPad Software, San Diego, CA, USA).

## 5. Conclusions

The present study demonstrates that *Sechium* hybrid H387 extract inhibits proliferation and induces apoptosis in K562 chronic myeloid leukemia cells. These effects were accompanied by reduced SIRT1 expression and nuclear localization and increased p53 expression and acetylation at lysine 382, supporting the involvement of the SIRT1–p53 regulatory axis in the cellular response to the extract. The phytochemical composition previously characterized for H387 provides a chemical context for the observed biological activity; however, the present data do not establish that any individual constituent is responsible for the observed effects or that SIRT1 is a direct molecular target of the extract. Importantly, these findings are limited to an in vitro K562 cell model and should not be interpreted as evidence of therapeutic efficacy. Further studies using purified or fractionated constituents, additional leukemia models, and appropriate in vivo investigations are required to establish the reproducibility, mechanism, biological relevance, and potential translational significance of H387 extract.

## Figures and Tables

**Table 1:** Major identified phytochemical constituents of *Sechium hybrid* H387 07 extract.

Compound	Chemical Class	Concentration (mg/g Extract)	Analytical Method	Reference
Galangin	Flavonoid	21.940	HPLC	[26]
Phloretin	Flavonoid	4.616	HPLC	[26]
Naringenin	Flavonoid	3.304	HPLC	[26]
Chlorogenic acid	Phenolic acid	4.224	HPLC	[26]
Cucurbitacin B	Cucurbitacin	4.595	HPLC/HPTLC	[27]
Cucurbitacin I	Cucurbitacin	3.061	HPLC/HPTLC	[27]
Cucurbitacin E	Cucurbitacin	0.594	HPLC/HPTLC	[27]

Note: Concentrations correspond to the quantified content reported for the H387 07 extract in the cited phytochemical studies [26,27].

## Data Availability

The raw data supporting the conclusions of this article will be made available by the authors on request.

## Abbreviations

The following abbreviations are used in this manuscript:

AML	Acute Myeloid Leukemia
CML	Chronic Myeloid Leukemia
TKI	Tyrosine Kinase Inhibitors
CP	Chronic Phase
AP	Accelerated Phase
BC	Blast Crisis
AcK382	Acetylation of p53 at lysine 382
IC	Inhibitory Concentration
